# Endoscopic Submucosal Dissection for Early Colorectal Carcinoma with High-risk Criteria: Long-term Follow-up from the German Multicenter Registry

**DOI:** 10.1055/a-2949-2685

**Published:** 2026-09-18

**Authors:** Sara Moser, Andreas Probst, Alanna Ebigbo, Ingo Steinbrück, Hans-Peter Allgaier, Jürgen Pohl, Roland Reinehr, Viktor Rempel, Michael Anzinger, Franz Ludwig Dumoulin, Jörg Schirra, Dani Dakkak, Ulrike Denzer, Andreas Wannhoff, Stefan Sulk, Siegbert Faiss, Anna Muzalyova, Carola Fleischmann, Helmut Messmann

**Affiliations:** 1 Department of GastroenterologyUniversity Hospital AugsburgAugsburgBayernGermany; 2 Department of GastroenterologySt. Josef-HospitalBochumGermany; 3 Medical Department 219616 Evangelisches Diakoniekrankenhaus FreiburgFreiburgBaden-WürttembergGermany; 4 Department of Gastroenterology 38169 Asklepios Klinik BarmbekHamburgHHGermany; 5 Department of Gastroenterology 14917 Asklepios Klinik AltonaHamburgHHGermany; 6 Department of GastroenterologyElbe-Elster Klinikum HerzbergHerzbergGermany; 7 Department of GastroenterologySt. Anna Hospital HerneHerneGermany; 8 Department of GastroenterologyKrankenhaus Barmherzige Brüder MünchenMünchenBayernGermany; 9 Department of GastroenterologyGemeinschaftskrankenhaus BonnBonnGermany; 10 Medical Department IIUniversity Hospital, Ludwig Maximilians-UniversityMunichGermany; 11 Department of Gastroenterology 14922 Elisabeth-Krankenhaus-EssenEssenGermany; 12 Department of GastroenterologyUniversity Hospital MarburgMarburgHEGermany; 13 Department of Gastroenterology 363255 Klinikum LudwigsburgLudwigsburgBWGermany; 14 Department of Gastroenterology 39063 University Hospital Carl Gustav CarusDresdenGermany; 15 Department of Gastroenterology 40656 Sana Klinikum LichtenbergBerlinBerlinGermany; 16 Institute of Digital Medicine 39694 University Hospital of AugsburgAugsburgBayernGermany; 17 Department of Gastroenterology, Hepatology and Endocrinology 9211 Klinikum NürnbergNuremberg NürnbergBayernGermany

**Keywords:** pT1 colorectal cancer, endoscopic submucosal dissection, risk stratification, additional surgery

## Abstract

**Background and Study Aim** Endoscopic submucosal dissection (ESD) is recommended for selected colorectal lesions, especially those with superficial submucosal invasion, enabling en-bloc resection and accurate histopathologic assessment. However, the management of high-risk lesions following ESD, especially regarding additional surgery versus surveillance, remains debated. This study evaluated outcomes after ESD for pT1 colorectal carcinoma, comparing surgical versus conservative management.

**Patients/Materials and Methods** This retrospective multicenter study included 92 patients with pT1 colorectal carcinoma treated by ESD at 14 German referral centers (January 2017–December 2020). Patients were classified as low-risk ( *n* = 34) or high-risk ( *n* = 58) based on histopathologic criteria. Long-term outcomes were compared between low- and high-risk patients, as well as between high-risk patients undergoing additional surgery versus conservative management.

**Results** The overall three-year disease-free survival was 82.6%, without a significant difference between the low-risk (88.2%) and high-risk groups (79.3%; *p* = 0.276). Among high-risk patients, 56.9% received additional surgery and 43.1% were managed conservatively. Recurrences occurred exclusively in the high-risk group (5.2%). The R0 resection rate after ESD was higher in patients managed without additional surgery than in those who subsequently underwent surgery (76.0% vs. 45.5%; *p* = 0.019).

**Conclusion** ESD is effective for curative management of selected early colorectal cancers (pT1), particularly in low-risk cases. In high-risk patients, surgery remains the standard, with no recurrences observed after additional surgery in our cohort. A substantial proportion of high-risk patients did not undergo surgery in clinical practice and showed a recurrence rate of 12% (3/25). These findings support current recommendations while reflecting real-world treatment patterns.

## Introduction

Endoscopic resection (ER) is recommended for colorectal lesions with suspected superficial submucosal invasion.

Particularly in the rectum, endoscopic submucosal dissection (ESD) is recommended. 1 ESD enables en-bloc resection, which is essential for accurate histopathologic assessment and thereby crucial for guiding further treatment and determining patient prognosis. 2 Compared to conventional endoscopic mucosal resection (EMR), ESD allows the complete resection of larger and fibrotic lesions in a single piece, improving both diagnostic accuracy and local disease control. 3 4

In cases of submucosal invasive colorectal cancer (SMIC) the risk of lymph node metastases (LNM) has to be defined according to histopathologic criteria after ER. In current guidelines, high-risk features are defined as submucosal invasion exceeding 1000 μm, lymphovascular invasion (LVI), poor differentiation (G3/4), high-grade tumor budding, or positive vertical resection margins. 5 6

In contrast, lesions fulfilling low-risk criteria (submucosal invasion <1000 μm, absence of LVI, G1/2, R0, no budding), the risk of LNM is negligible, ER may be considered curative, and additional surgery is generally not required. 1

Conversely, the presence of high-risk histologic features or a positive vertical margin indicates a noncurative high-risk resection and usually warrants further surgical treatment. In contrast, a positive horizontal margin in an otherwise low-risk lesion is considered a local-risk resection, mainly associated with an increased risk of local recurrence. 1 7 8

Regarding the depth of submucosal invasion, current evidence suggests that deep submucosal invasion (DSI) alone is associated with a relatively low LNM risk of only 2.6%, calling into question the necessity of surgical intervention based solely on this parameter. 9

Furthermore, the existing body of evidence on ESD for pT1 colorectal carcinoma (CRC) is predominantly derived from Asian studies. Large-scale, multicenter data from Western populations are still lacking, limiting the generalizability of current treatment recommendations.

It should be noted, however, that surgical resection is not always feasible, as certain cases may preclude surgery due to significant comorbidities, patient preference, or individualized clinical decision-making by the treating physician. 10 11

The aim of this study was to evaluate long-term survival outcomes in patients with high-risk early colorectal cancer (pT1a) treated with ESD, comparing those who underwent additional surgery with those who did not receive further surgical intervention.

## Patients/Material and Methods

This multicenter study included patients with CRC who underwent ESD between January 2017 and December 2020 at 14 referral centers participating in the German ESD registry. The dataset included a total of 819 patients who underwent ESD for colorectal lesions. This study is based on a retrospective data analysis.

The German ESD registry was initiated by the University Hospital of Augsburg and was approved by the ethics committee of the Ludwig-Maximilian-University, Munich (study identifier: DRKS00011781).

For the present analysis, only patients with histologic evidence of SMIC in the ESD specimen were included. Patients were stratified into low-risk and high-risk groups based on established histopathologic criteria.

Low-risk lesions were defined as tumors with submucosal invasion depth ≤1000 μm, absence of lymphovascular invasion (L0, V0), good or moderate differentiation (G1/G2), and absence or low-grade tumor budding (BD1). High-risk lesions were defined by the presence of at least one of the following criteria: submucosal invasion depth >1000 μm, presence of LVI (L1 or V1), poor differentiation (G3), high-grade tumor budding (BD2 or BD3) or positive vertical and horizontal resection margin.

Patient demographics, lesion characteristics, procedural details, histopathologic findings, and clinical outcomes were retrieved from the ESD registry database. The decision for additional surgical treatment following ESD was made individually by the treating physicians based on guideline recommendations, patient comorbidities, and shared decision-making.

The study was conducted in accordance with the principles of Good Clinical Practice and the ethical guidelines of the 1975 Declaration of Helsinki.

### Inclusion Criteria

Patients were included in the study if they had a histologically confirmed pT1 adenocarcinoma of the colon or rectum resected via ESD, had provided written informed consent for the ESD procedure after receiving comprehensive information about the technique and available alternative treatment options, and had given written informed consent for inclusion in the German ESD registry database. A total of 92 patients met all inclusion criteria and were enrolled in the final analysis ( Fig. 1 ).

**Fig. 1:**
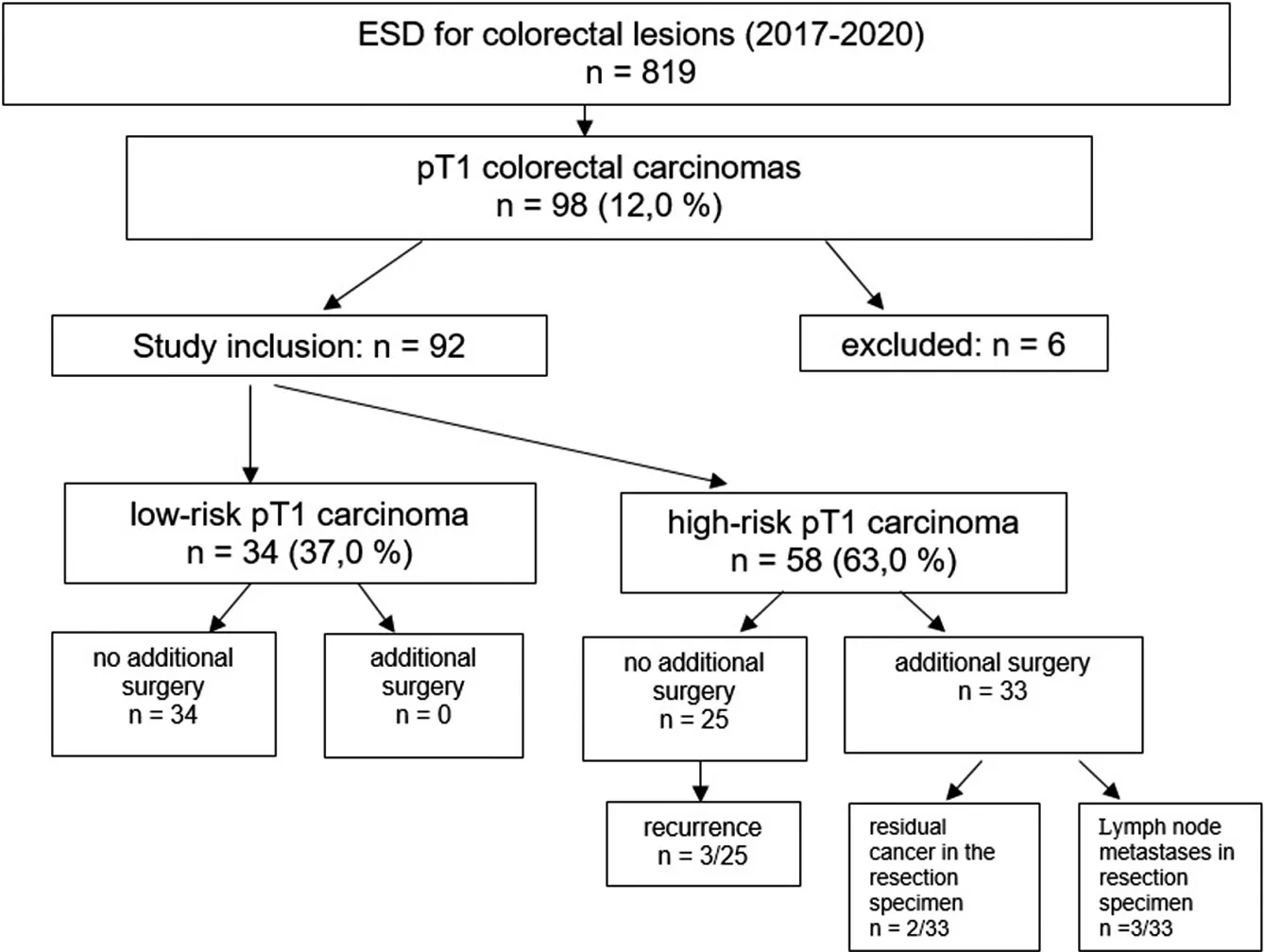
Patient inclusion.

### Exclusion Criteria

Patients were excluded from the analysis if follow-up data were unavailable (lost to follow-up, *n* = 6) ( Fig. 1 ).

### Statistical Analysis

Categoric variables were summarized as absolute frequencies and percentages. Comparisons between groups were performed using the Chi-square test or Fisher’s exact test, as appropriate.

Interval-scaled variables were described using medians and interquartile ranges (IQRs). Between-group comparisons for these variables were conducted using the Mann–Whitney *U* test as data were not normally distributed and group sizes were unequal.

Survival analysis was performed using Kaplan–Meier curves to estimate time-to-event outcomes. Differences between groups were assessed using the log-rank test.

A *p* -value of < 0.05 was considered statistically significant. Statistical analyses were performed using IBM SPSS Statistics (Version 31.0.2.0, IBM Corp., Armonk, NY, USA).

No adjustment for multiple statistical testing was performed.

Competing risks were analyzed using Fine–Gray subdistribution hazard models to account for the presence of competing events, using the cmprsk package in R. The event of interest was defined as colorectal cancer recurrence or colorectal cancer-related death, whereas death from other causes was considered a competing event. Subdistribution hazard ratios (sHRs) with corresponding 95% confidence intervals were estimated to assess the association between treatment group and the cumulative incidence of the event of interest in the presence of competing events.

### Diagnostic Workup and ESD Procedure

Diagnostic endoscopy and the ESD procedure were performed at the different centers at the discretion of the local endoscopist. ESD was selected in cases where en-bloc resection was unlikely to be achieved using conventional techniques such as EMR, particularly for colorectal lesions larger than 20 mm, fibrotic lesions, or those with nonlifting signs. Lesion morphology was classified according to the Paris classification. 12

There was no uniform protocol for baseline staging prior to or following endoscopic resection. Additional diagnostic procedures such as endoscopic ultrasound (EUS), computed tomography (CT), or magnetic resonance imaging (MRI) were conducted based on individual clinical judgment and the recommendations of the local multidisciplinary team. Follow-up data were collected retrospectively from all participating centers.

## Results

### Patient and Lesion Characteristics

A total of 92 patients were included, with 34 classified as low-risk and 58 as high-risk based on histopathologic criteria. The groups were comparable regarding age, sex, and ASA status.

The majority of lesions were located in the rectum. The maximum lesion diameter was comparable between the two groups. Nearly 95% of high-risk patients exhibited DSI (≥1000 μm), with a subset also demonstrating lymphatic (L1) and/or vascular (V1) invasion ( Table 1 ).

**Table 1:** Patient and lesion baseline characteristics, baseline staging procedures, specimen characteristics.

Characteristic	All patients ( *n* = 92)	Low-risk ( *n* = 34)	High-risk ( *n* = 58)	*p* Value
**Patient characteristics**				
*Age, y*	68 [56–76]	64 [55–76]	69 [56.75–78]	*p* = 0.346
*Sex*				
Female	32	16 (47.16%)	16 (27.59.0%)	
Male	60	18 (52.94.0%)	42 (72.41%)	*p* = 0.058
*ASA status I/II/III/IV*	36/41/13/2	13/15/6/0	23/26/7/2	*p* = 0.749
		(38.24%/44.12%/17.65%/0.00%)	(39.66%/44.83%/12.07%/3.45%)	
**Location**				
*Colon*	17	7 (20.0%)	10 (17.24%)	
*Rectum*	75	27 (80.0%)	48 (82.76%)	*p* = 0.904
*Colon*	17	7	10	
Cecum	2	1 (14.3%)	1 (10.0%)	
Ascending colon	1	1 (14.3%)	0 (0.0%)	
Transverse colon	2	1 (14.3%)	1 (10.0%)	
Descending colon	2	1 (14.3%)	1 (10.0%)	
Sigmoid	10	3 (42.9%)	7 (70.0%)	*p* = 0.758
Rectum	75	27	48	
Proximal 12–16	4	1 (3.7%)	3 (6.3%)	
Middle 6–12	31	13 (48.1%)	18 (37.5%)	
Distal 0–6	40	13 (48.1%)	27 (56.3%)	*p* = 0.616
**Lesion characteristics**				
Max. diameter, mm	34.50 [20–50]	38.5 [24.75–50]	29 [17.75–49.25]	*p* = 0.291
**Paris classification**				
0-Ip	3	2 (5.88%)	1 (1.72%)	
0-Ip + IIa	1	1 (2.94%)	0 (0.0%)	
0-Is	17	4 (11.76%)	13 (22.41%)	
0-Is + IIa	9	2 (5.88%)	7 (12.07%)	
0-Is + IIc	3	2 (5.88%)	1 (1.72%)	
0-IIa	36	12 (35.29%)	24 (41.38%)	
0-IIa + IIb	13	9 (26.47%)	4 (6.90%)	
0-IIa + IIc	5	1 (2.94%)	4 (6.90%)	
0-IIb	1	0 (0.0%)	1 (1.72%)	
0-IIb + IIc	1	0 (0.0%)	1 (1.72%)	
0-IIc	2	1 (2.94%)	1 (1.72%)	
0-III	0	0 (0.0%)	0 (0.0%)	
Missing information	1	0 (0.0%)	1 (1.72%)	*p* = 0.246
**Histopathology**				
*Depth of invasion*				
sm <1000 μm	37	34 (100.0%)	3 (5.17%)	
sm ≥ 1000 μm	55	0 (0.0%)	55 (94.83%)	p > 0.01
sm ≥ 1000 μm as Single risk factor	26	0 (0.0%)	26 (44.83%)	p > 0.01
Grading 1/2/3	28/61/3	20/14/0	8/47/3	p < 0.01
		(58.82%/41.18%/0.00%)	(13.79%/81.03%/5.17%)	
L 0/1	77/15	34/0 (100.00%/0.00%)	43/15 (74.14%/25.86%)	p < 0.01
V 0/1	85/7	34/0 (100.00%/0.00%)	51/7 (87.93%/12.07%)	*p* = 0.044
**BD > 1**	3	0 (0.00%)	3 (5.17%)	
**Baseline staging**				
None	52	19 (55.88%)	33 (56.90%)	*p* = 1.000
EUS	29	13 (22.41%)	16 (42.11%)	*p* = 0.354
MRT	21	5 (13.16%)	16 (42.11%)	*p* = 0.202
CT	14	1 (2.94%)	13 (22.41%)	*p* = 0.015
Abdominal ultrasound	1	0 (0.00%)	1 (1.72%)	*p* = 1.000
**Resection characteristics**				
Maximal diameter of the resection specimen, mm	42 [30–65]	41.5 [30.75–62.75]	42 [30.0–69.25]	*p* = 0.792
R0 resection	71	34 (100.0%)	37 (63.79%)	p < 0.01
R1 resection	21	0 (0.00%)	21 (36.21%)	p < 0.01
HM	9	0 (0.00%)	9 (15.52%)	
VM	12	0 (0.00%)	12 (20.70%)	

ASA: American Society of Anesthesiologists. HM: horizontal margin, VM: vertical margin, BD: tumor budding grade.

### Resection Characteristics

The mean resection specimen size was comparable between groups. R1 resections (positive horizontal or vertical margins) occurred in the high-risk group in 36.2 % of the patients ( Table 1 ).

### Baseline Staging

Pre-interventional imaging (CT, MRI, EUS) was more frequently performed in the high-risk group ( Table 1 ).

### Characteristics of High-risk pT1 Colorectal Carcinoma Patients with and without Additional Surgery

Among the 58 high-risk pT1 CRC patients, 33 underwent additional surgical resection. Demographic, histopathologic, and procedural characteristics were compared between high-risk patients with and without additional surgery.

Patients in the nonsurgical group were slightly older compared to those who received additional surgery. ASA classification was similar between the groups.

DSI (≥1000 μm) was present in nearly all patients in both groups. Grading and vascular invasion (V1) were evenly distributed across both groups, while lymphatic invasion (L1) was more frequent among surgically treated patients. A significantly higher proportion of patients in the nonsurgical group achieved R0 resection.

In the surgical group, 6 lesions (18.2%) were located in the colon and 27 (81.8%) in the rectum. Corresponding surgical procedures included total mesorectal excision for lower rectal lesions, low anterior resection for middle rectal lesions, and anterior resection for upper rectal lesions. Colonic lesions were treated with oncologic segmental resections, including sigmoid resection, left hemicolectomy, or right hemicolectomy, depending on tumor location.

In the nonsurgical group, 4 lesions (16.0%) were located in the colon and 21 (84.0%) in the rectum. Notably, 66.7% (14/21) of rectal cancers in this group were located in the lower rectum ( Table 2 ).

**Table 2:** Subgroup analysis of high-risk lesions.

Characteristic of high-risk pT1 colorectal carcinoma	Additional surgery *n* = 33	No additional surgery *n* = 25	*p* Value
**Patient characteristics**			
Age, y	68[55.5–76.5]	71[59.5–79.5]	*p* = 0.206
ASA status I/II/III/IV	13 (39.4%)/14(42.4%)/5(15.2%)/1(3.0%)	10(40.0%)/12(48.0%)/2(8.0%)/1(4.0%)	*p* = 0.904
**Location**			
*Colon*	6 (18.2%)	4 (16.0%)	
*Rectum*	27 (81.8%)	21 (84.0%)	
*Colon*	6	4	
Cecum	1 (16.7%)	0	
Ascending colon	0	0	
Transverse colon	0	1 (25.0%)	
Descending colon	1 (16.7%)	0	
Sigmoid	4 (66.6%)	3 (75.0%)	
*Rectum*	27	21	
Proximal 12–16	1 (3.8%)	2 (9.5%)	
Middle 6–12	13 (48.1%)	5 (23.8%)	
Distal 0–6	13 (48.1%)	14 (66.7%)	
**Histopathology**			
*Depth of invasion*			
sm < 1000 μm	1 (3.03%)	0 (0.0%)	
sm ≥ 1000 μm	32 (96.97%)	25 (100.0%)	*p* = 1.00
*sm ≥ 1000 μm as Single risk factor*	10 (30.30%)	16 (64.0%)	*p* = 0.011
*Grading 1/2/3*	5(15.2%)/26(78.8%)/2(6.1%)	3(12.0%)/21(84.0%)/1(4.0%)	*p* = 1.00
*L 0/1*	21(63.6%)/12(36.4%)	22 (88.0%)/3(12.0%)	*p* = 0.036
*V 0/1*	29(87.9%)/4(12.1%)	22 (88.0%)/3(12.0%)	*p* = 1.00
BD>1	3	0	
**Reason for conservative approach**			
Comorbidities		5 (20%)	
Physician decision		9 (36%)	
Patient refusal		11 (44%)	
**Resection characteristics**			
*R0 resection*	18 (54.54%)	19 (76.0%)	*p* = 0.107
*R1 resection*	15 (45.45%)	6 (24.0%)	
HM	6(40.0%)	3 (50.0%)	
VM	9(60.0%)	3 (50.0%)	
**Recurrence, n**	0	3	
Endoluminal	0	1	
Extraluminal	0	1	
Distant metastasis	0	1	
**Death, n**	4 (12,12%)	8 (32,0%)	
Death by colorectal cancer	0	1	

ASA: American Society of Anesthesiologists. HM: horizontal margin, VM: vertical margin.

### Histopathologic Findings in Surgical Resection Specimens after ESD

Among high-risk patients who underwent completion surgery after ESD, lymph-node metastases were identified in three cases. In the first case, the index ESD had been R1 at the VM with lymphovascular invasion (L1) and moderate differentiation (G2); 2/20 lymph nodes were positive, but no residual primary tumor was present. In the second, the index lesion showed submucosal invasion >1000 μm with BD3, L1, G2, and a R0 resection; 2/22 nodes were positive with no intramural residual tumor. The third node-positive case was an index lesion characterized by DSI as the sole risk factor (R0, L0, V0); the surgical specimen showed pN1 with 1/24 nodes positive and no residual primary tumor. All three lesions were located in the rectum.

Residual carcinoma was identified in two additional surgical specimens; both corresponding index ESDs had been R1 at the HM with negative vertical margin (VM), G2, L0, V0 – one with a DSI of 1000 μm and the other with DSI of 2000 μm.

In the high-risk cohort undergoing completion surgery, 15 index ESD specimens were R1; however, residual tumor was detected in only 2 corresponding surgical specimens (13.3%) ( Table 2 ).

### Follow-up after ESD

During follow-up, a total of 66 patients (71.7%) underwent at least one restaging procedure. Endoscopic surveillance was the most frequently performed measure. The mean number of endoscopies was significantly higher in the high-risk group compared to the low-risk group. Other follow-up procedures as EUS, CT and MRI were performed ( Table 3 ).

**Table 3:** Follow-up after ESD.

Further course after ESD	All patients ( *n* = 92)	Low-risk ( *n* = 34)	High-risk ( *n* = 58)	*p* Value
**Course during FU**				
Staging procedures during FU				
*Endoscopies*	66	21 (61.7%)	41 (70.7%)	*p* = 0.378
No. of endoscopies	3.34 SD = 2.155 (0–7)	2.59 SD = 1.617 (0–7)	3.78 SD = 2.317 (0–7)	*p* = 0.025
*EUS*	4	1 (2.9%)	3 (5.2%)	*p* = 0.612
*CT scans/MRI*	12	2(5.9%)	10(17.24%)	*p* = 0.118
**Recurrence, n**	3	0 (0.0%)	3(5.2%)	*p* = 0.293
Endoluminal	1	0(0.0%)	1 (33.3%)	
Extraluminal	1	0(0.0%)	1 (33.3%)	
Distant metastasis	1	0(0.0%)	1 (33.3%)	*p* = 1.00
**Death, n**	17 (18.5%)	5 (14.7%)	12 (21.1%)	*p* = 0.452
Death by colorectal cancer	1 (1.1%)	0 (0.0%)	1 (8.3%)	*p* = 0.515
**Survival %**				
Overall survival at 3 years, %	77 (83.7%)	30 (88.2%)	47(81.0%)	*p* = 0.367
Disease-free survival at 3 years, %	71(82.6%)	30(88.2%)	46(79.3%)	*p* = 0.276
FU (Exitus), months	70.8 (CI:63.8;77.9)	64.7 (CI:53.4;76.0)	72.1 (CI:63.6;81.6)	
FU DFS, months	71.2 (CI 64.7–77.7)	68.6 (CI:59.2–78.1)	72.7 (CI:63.7–81.7)	

FU: follow up. EUS: endoscopic ultrasound. CT: computed tomography. MRI: magnetic resonance imaging. DFS: disease-free survival. CI: confidence interval.

### Recurrence and Metastasis

Tumor recurrence was observed in three patients (3.3%), all of whom were in the high-risk group without additional surgery. No recurrence was detected among low-risk patients ( Table 3 ).

One case each of endoluminal, extraluminal, and distant metastatic recurrence was documented. All three recurrence cases occurred in patients with lower rectal lesions located 0–3 cm from the anal verge.

Histopathologic analysis of the initial ESD specimens in all three recurrence cases revealed DSI (>1.000 μm). One patient had an L0/V1 status with an R1 resection at the horizontal margin. Surgery was recommended, but the patient declined; instead, an EMR of the scar was performed, which showed no residual tumor histologically. However, an endoluminal recurrence was detected 9 months later. The patient then consented to surgery (low anterior rectal resection), which confirmed detection of tumor tissue in the resection specimen but no lymph node metastases. The further course was uneventful.

A second patient had an L0/V0 status, with DSI of 1,500 μm as the only risk factor. Due to an individualized decision by the treating physician, no surgery was performed after ESD. An extraluminal recurrence occurred 4 years later, after which the patient underwent surgical resection. The subsequent course was without further events.

The third recurrence involved a patient with L1/V1 status and R0 resection. This patient initially refused additional surgery. Two years after ESD, the patient developed hepatic and peritoneal metastases and subsequently received palliative chemotherapy but died one year after the diagnosis of metastatic recurrence.

The distant metastasis rate within the high-risk group was 1.7% (1/58).

### Mortality and Cause of Death

About 18.5% of the patients died during the follow-up period. Only one death was attributed to colorectal cancer, occurring in the high-risk group ( Table 3 ).

### Survival Outcomes

At three years, overall survival was 83.7% for the cohort (88.2% in the low-risk and 81.0% in the high-risk strata), while disease-free survival (DFS) was 82.6% overall, with rates of 88.2% and 79.3% in the low- and high-risk groups, respectively ( Table 3 , Figs. 2 and 3 ).

**Fig. 2:**
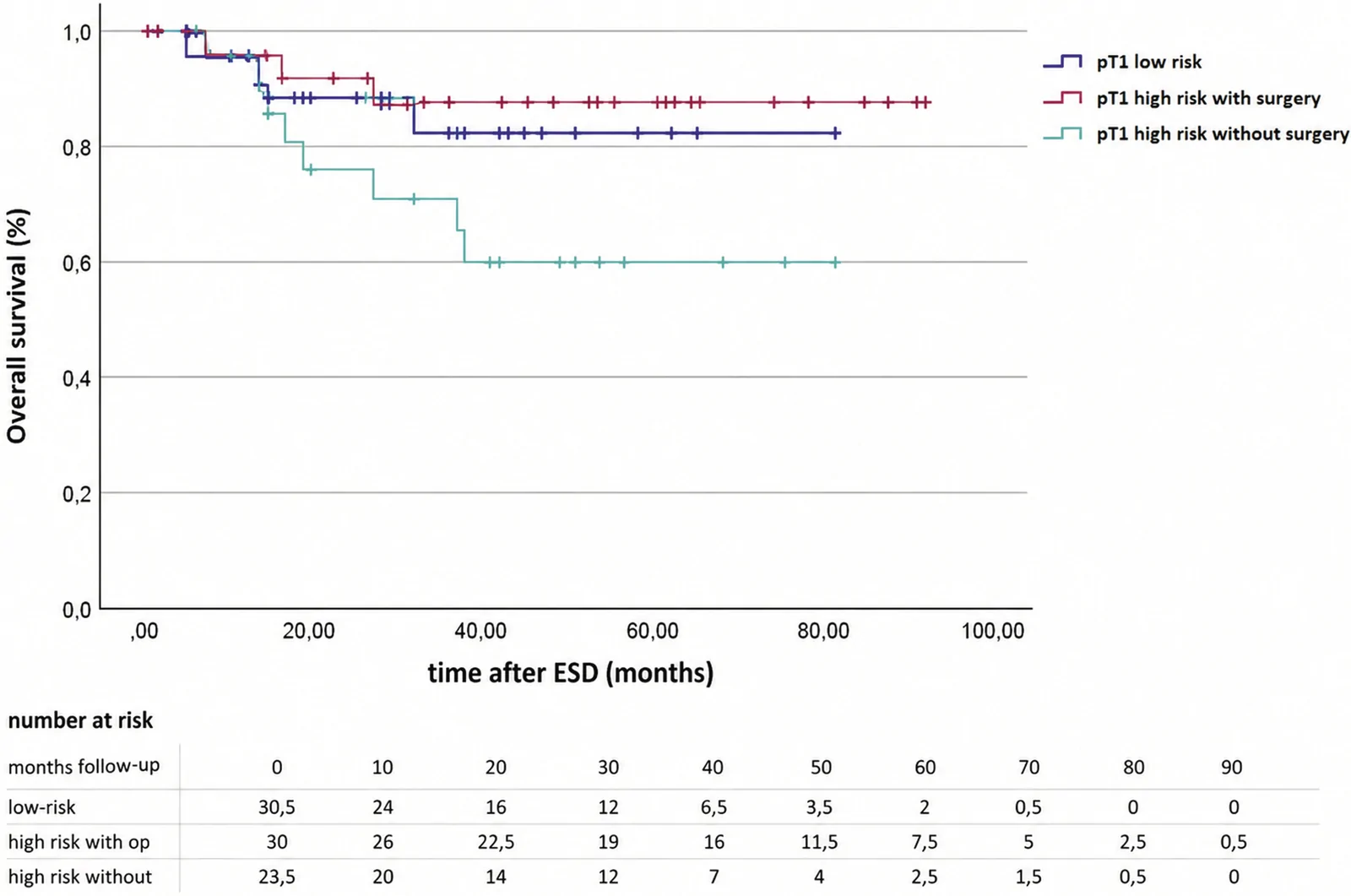
Kaplan-Meier analysis of overall survival after ESD for pT1 colorectal cancer (stratified by risk group), including number at risk.

**Fig. 3:**
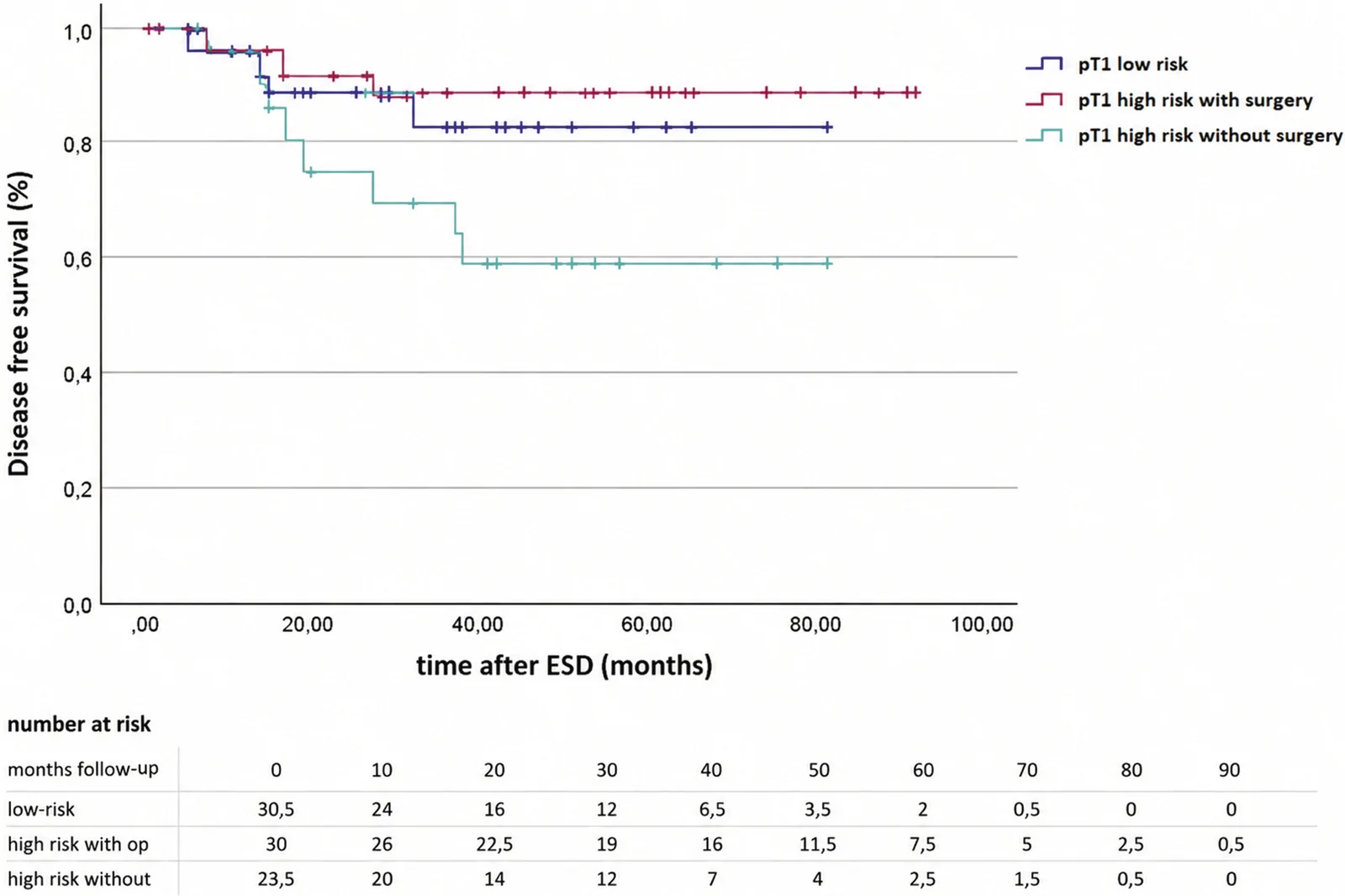
Kaplan-Meier analysis of disease-free survival after ESD for pT1 colorectal cancer (stratified by risk group), including number at risk.

In the Fine–Gray competing-risk analysis, with colorectal cancer recurrence or colorectal cancer-related death as the event of interest and death from other causes as the competing event, neither high-risk patients who underwent additional surgery (sHR 0.66, 95% CI 0.15–2.94; *p* = 0.59) nor high-risk patients without additional surgery (sHR 2.11, 95% CI 0.63–7.09; *p* = 0.22) differed significantly from the low-risk reference group. The overall group effect was not statistically significant.

### Follow-up Duration

The mean follow-up duration was 70.8 months for the overall cohort. Patients in the low-risk group had a mean follow-up of 64.7 months, compared to 72.1 months in the high-risk group ( Table 3 ).

## Discussion

This multicenter retrospective study evaluated the long-term outcomes of patients with early CRC (pT1) treated by ESD, with a particular focus on high-risk patients who did or did not undergo additional surgery. The findings contribute valuable real-world data on the safety and oncologic adequacy of ESD, particularly in settings where additional surgery is not feasible or declined.

The overall three-year DFS and overall survival (OS) rates of 82.6% and 83.7%, respectively, are comparable to previous reports and support the role of ESD as a viable treatment option in appropriately selected patients with early colorectal cancer. The DFS was slightly higher in the low-risk group (88.2%) than in the high-risk group (79.3%), although this difference did not reach statistical significance. In the high-risk cohort treated surgically, the decline in overall survival represents a concentrated occurrence of mortality events early in the follow-up period, which may be attributable to the patients’ increased comorbidity burden and the elevated perioperative risk profile inherent to this subgroup.

The findings were supported by a Fine–Gray competing-risk analysis accounting for deaths unrelated to colorectal cancer, which likewise demonstrated no statistically significant differences in the cumulative incidence of colorectal cancer recurrence or colorectal cancer-related death between the study groups. However, the small number of oncologic events limits the statistical power.

Importantly, all tumor recurrences were observed in the high-risk group, reinforcing the relevance of histopathologic risk stratification for postoperative decision-making.

Among high-risk patients, additional surgery was performed in 33 cases (56.9%), whereas 25 patients (43.1%) did not undergo further surgical intervention.

Comparison of these two groups revealed that patients managed conservatively were older and more likely to have DSI as the sole risk factor (DSI as only risk factor in 64.0% vs. 30.3%; *p* = 0.011), while features such as lymphatic invasion and positive margins were significantly more frequent in the surgical group, indicating a greater inclination toward surgery when this high-risk feature was present. Interestingly, deep invasion as the only risk factor was significantly more common in the nonsurgical group, suggesting that the absence of additional risk features may have influenced the decision to forgo surgery. Tumor location, particularly in the lower rectum, may have influenced treatment decisions due to the higher morbidity associated with surgical resection in this region. These findings reflect current clinical practice, where individualized decisions—often influenced by age, comorbidities, and patient preference—guide the choice of further treatment.

Interestingly, lymph node metastases were found in only 3 out of 33 patients who underwent surgery (9.1%), and residual tumor tissue was present in 2 cases (6.1%). While this highlights the potential for residual disease, it also underscores that a significant proportion of high-risk patients may not harbor further pathologic findings despite meeting criteria for additional surgery. The recurrence rate in the nonsurgical high-risk group was 12.0% (3/25), including one fatal metastatic event. These outcomes emphasize the need for a risk-adapted approach and rigorous post-ESD surveillance, particularly for patients who forgo surgery.

The relatively high R0 resection rate in the nonsurgical high-risk group (76.0% vs. 54.5% in the surgical group) likely reflects a selection bias, with patients showing favorable resection margins being managed more conservatively. This aligns with recent literature suggesting that ESD with R0 resection and limited risk features (e.g., deep invasion without lymphovascular invasion or poor differentiation) may be sufficient in selected cases. 13 14

Follow-up intensity differed significantly between groups: high-risk patients underwent a greater number of surveillance endoscopies (mean 3.78 vs. 2.59; *p* = 0.025), suggesting more cautious monitoring where surgical therapy was deferred. Despite this, recurrence still occurred exclusively in the high-risk group, supporting guideline recommendations for careful long-term follow-up in these patients.

Only one patient (1.1%) died of colorectal cancer during the follow-up period, which speaks of the generally favorable prognosis of early pT1 CRC when appropriately treated. Most deaths were unrelated to cancer, highlighting the importance of balancing oncologic risk with overall patient health status when making treatment decisions.

This study has several strengths, including its multicenter design, inclusion of a well-characterized patient cohort, and detailed follow-up data. However, limitations must be acknowledged. The retrospective design introduces potential selection and information biases. In addition, staging procedures were not performed systematically in all high-risk patients and were left to the discretion of the treating centers, which is not fully in line with current guideline recommendations. Follow-up protocols were not standardized, and the decision for surgery was not randomized, potentially affecting outcomes. Additionally, the relatively small number of events (recurrences, cancer-related deaths) limits the statistical power to detect survival differences.

The classification of margin status in this study represents a limitation. Horizontal and vertical margin involvement were grouped together within the high-risk category, although current guidelines distinguish between local-risk (horizontal margin) and noncurative high-risk (vertical margin) resections. This may have influenced risk stratification and outcome interpretation.

In conclusion, our findings support the use of ESD as a curative-intent therapy for pT1 CRC in selected patients. In high-risk cases, additional surgery should remain the standard of care; however, in selected patients with favorable features or contraindications to surgery, conservative management after ESD with close surveillance may be considered in selected patients who are unfit for surgery or decline surgical treatment. Further prospective studies are warranted to refine risk stratification models and define optimal surveillance strategies for patients with high-risk histopathologic features who do not undergo additional resection.

**Abbreviations**
ER: Endoscopic resection
ESD: endoscopic submucosal dissection
EMR: endoscopic mucosal resection
SMIC: submucosal invasive colorectal cancer
LNM: lymph node metastases
LVI: lymphovascular invasion
DSI: deep submucosal invasion
CRC: colorectal carcinoma
EUS: endoscopic ultrasound
CT: computed tomography
MRI: magnetic resonance imaging
HM: horizontal margin
VM: vertical margin
BD: tumor budding grade
DFS: disease-free survival
CI: confidence interval
sHR: subdistribution hazard ratio
